# Supplementary material for: Comparison of Escherichia coli surface attachment methods for single-cell microscopy
Source: Sci Rep. 2019 Dec 19;9:19418. doi: 10.1038/s41598-019-55798-0 (PMC6923479; doi:10.1038/s41598-019-55798-0)
Supplement: Supplementary file 8 — Supplementary information8 [file 41598_2019_55798_MOESM8_ESM.pdf]

Supplementary Information for "Comparison of *Escherichia coli* surface attachment methods for single-cell microscopy"

Yao-Kuan Wang[1,+], Ekaterina Krasnopeeva[2,+], Ssu-Yuan Lin[1], Fan Bai[3], Teuta Pilizota[2,*], and Chien-Jung Lo[1,*]

[1]Department of Physics and Graduate Institute of Biophysics, National Central University, Jhongli, Taiwan 32001, ROC
[2]Centre for Synthetic and Systems Biology, Institute of Cell Biology, School of Biological Sciences, University of Edinburgh, Alexander Crum Brown Road, EH9 3FF, Edinburgh, UK
[3]Biodynamic Optical Imaging Center (BIOPIC), School of Life Sciences, Peking University, Beijing 100871, China
[*]corresponding authors teuta.pilizota@ed.ac.uk, cjlo@phy.ncu.edu.tw
[+]these authors contributed equally to this work

Supplementary Materials

Supplementary Methods: pHluorin calibration

The *in vivo* calibration of pH sensor was performed as follows[1]. The mixture of 100 mM MES, HEPES and AMPSO buffers was adjusted to a set of pH values in the range between 5.5 and 9, and supplemented with one of the three pH collapsing agents: 40 mM potassium benzoate and 40 mM methylamine hydrochloride (PBMH)[2], 25 μM CCCP or 5 mM indole[3]. Tunnel-slides were prepared as previously described[1,4]: two bits of sticky tape form a tunnel and are sandwiched between a coverslip and a microscope slide. Buffer of known pH was flushed into a channel, incubated for 15 min, upon which 5 different fields of view containing over 100 cells were imaged with 50 ms exposure time. The calibration curves were plotted as ratio of emission intensities for excitation at 395 nm and 475 nm against pH, and fitted with the sigmoid function $R_{395/475} = (a_1 e^{k(pH-pH_0)} + a_2)/(e^{k(pH-pH_0)} + 1)$, where a_1, a_2, k and pH_0 are free fitting parameters.

In vitro calibration was performed with the purified pHluorin protein diluted into buffer of known pH in the 96-well plate (Thermo Scientific, Optical bottom). The pHluorin excitation spectra for 510 nm emission was measured in Spark 10M multimode plate reader (Tecan Trading AG, Switzerland). The His-tagged protein was purified using affinity chromatography column[1]. The excitation spectra was scanned from 380 nm to 480 nm with 5 nm step size. Additionally, the autofluorescence of the buffer with no added protein was measured and subtracted from the measured protein intensity.

Supplementary Videos

SI-Video 1: Time lapse movie of cells growth on the APTES surface, total time 10 hours.
SI-Video 2: Time lapse movie of cells growth on the PLL in-chamber surface, total time 12 hours.
SI-Video 3: Time lapse movie of cells growth on the PLL rinsed surface, total time 12 hours.
SI-Video 4: Time lapse movie of cells growth on the PLL air-dried surface, total time 12 hours.
SI-Video 5: Time lapse movie of cells growth on the PEI surface, total time 12 hours.
SI-Video 6: Time lapse movie of cells growth on the Cell-Tak surface, total time 12 hours.
SI-Video 7: Time lapse movie of cells growth on the Gel pad surface, total time 12 hours.

Supplementary Tables

Media and Strain	t_D at 37°C	t_D at 25°C	t_D at 23°C	Reference
Nutrient Broth, *Salmonella typhimurium*	25 min	56 min	—	[5]
199 Tissue Culture Medium, *Salmonella typhimurium*	32 min	68 min	—	[5]
0.2% Glucose Medium, *Salmonella typhimurium*	50 min	92 min	—	[5]
0.2% Succinate Medium, *Salmonella typhimurium*	65 min	125 min	—	[5]
0.2% Lactate Medium, *Salmonella typhimurium*	67 min	120 min	—	[5]
Rich Glucose Media, *E. coli* B/r NC3	33 min	—	100 min	[6]
Minimal Medium, *E. coli* B/r NC3	—	—	157 min	[6]
LB, *E. coli* K-12 MG1655	22 min	—	—	This work
MM9 Medium, *E. coli* K-12 MG1655	49 min	—	—	This work
LB, *E. coli* K-12 EK03	26 min	—	86 min	This work
MM9 Medium, *E. coli* K-12 EK03	58 min	—	210 min	This work

Table 1. Population doubling times at different temperatures ($t_D = ln(2)/\lambda$). Nutrient Broth (Meat extract +0.2% peptone +0.16% glucose and Salt solution), 199 Tissue Culture Medium (as in[7]), 0.2% Glucose Medium (0.2% glucose + Salt Solution), 0.2% Succinate Medium (0.2% succinate + Salt Solution), 0.2% Lactate Medium (0.2% lactate + Salt Solution), Salt Solution ($MgSO_4 \cdot 7H_2O$ – 0.1, citric acid – 1.0, $Na_2HPO_4 \cdot 2H_2O$ – 5.0, $Na(NH_4)HPO_4 \cdot 4H_2O$ – 1.74, KCl – 0.74 g/l), Minimal Medimum (defined MOPS medium[8]), Rich Glucose Medium (MOPS medium supplemented with 0.4% glucose, amino acids (minus leucine; 0.12 mM valine and 0.08 mM isoleucine), 0.01 mM of each of the five vitamins (p-aminobenzoic acid, p-dihydroxybenzoic acid, p-hydroxybenzoic acid, pantothenate (calcium salt), and thiamine) and 0.2 mM of each of four bases (adenine, guanine, cytosine, and uracil)).

Supplementary Figures

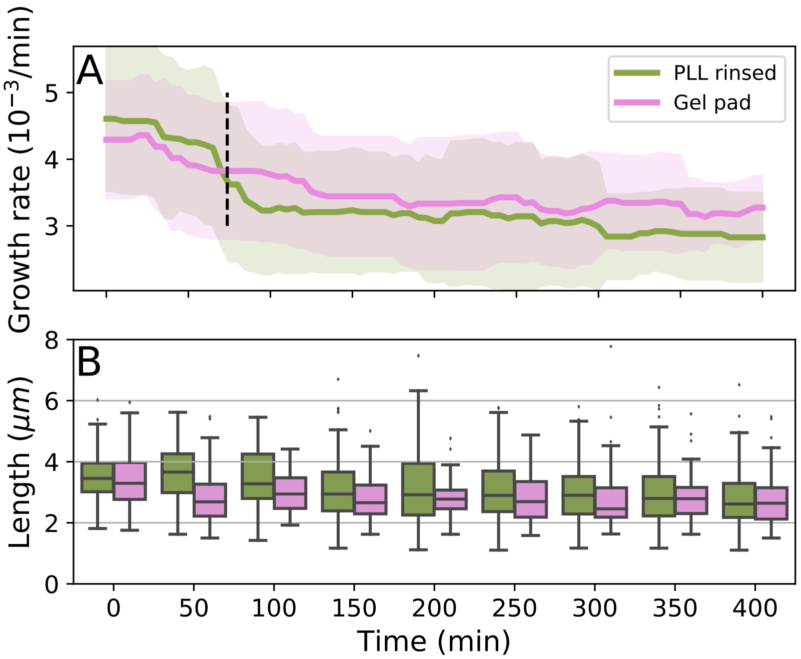

SI Figure 1. (**A**) Growth rate (b) decreases on PLL rinsed and gel pad surfaces during G_0 (see Figure 1, top). Dotted horizontal line at 73 min indicates completion of G_0 and beginning of G_1, after which the growth rate remains roughly constant. We attribute the reduction in growth rate during G_0 to adaptation to growth at 23°C after the culture has been grown at 37°C (see also *Materials and Methods*. (**B**) Cell length distribution over time on the PLL rinsed and a gel pad surfaces changes in line with growth rate changes in (A). The average length of the cells population decreases from 3.56 ± 0.81 µm to 2.75 ± 0.80 µm on the PLL rinsed, and from 3.41 ± 0.78 µm to 2.73 ± 0.81 µm on the gel pad. A steady length distribution is reached by G_1. Cells grow on the other tested surface showed similar length distribution over time.

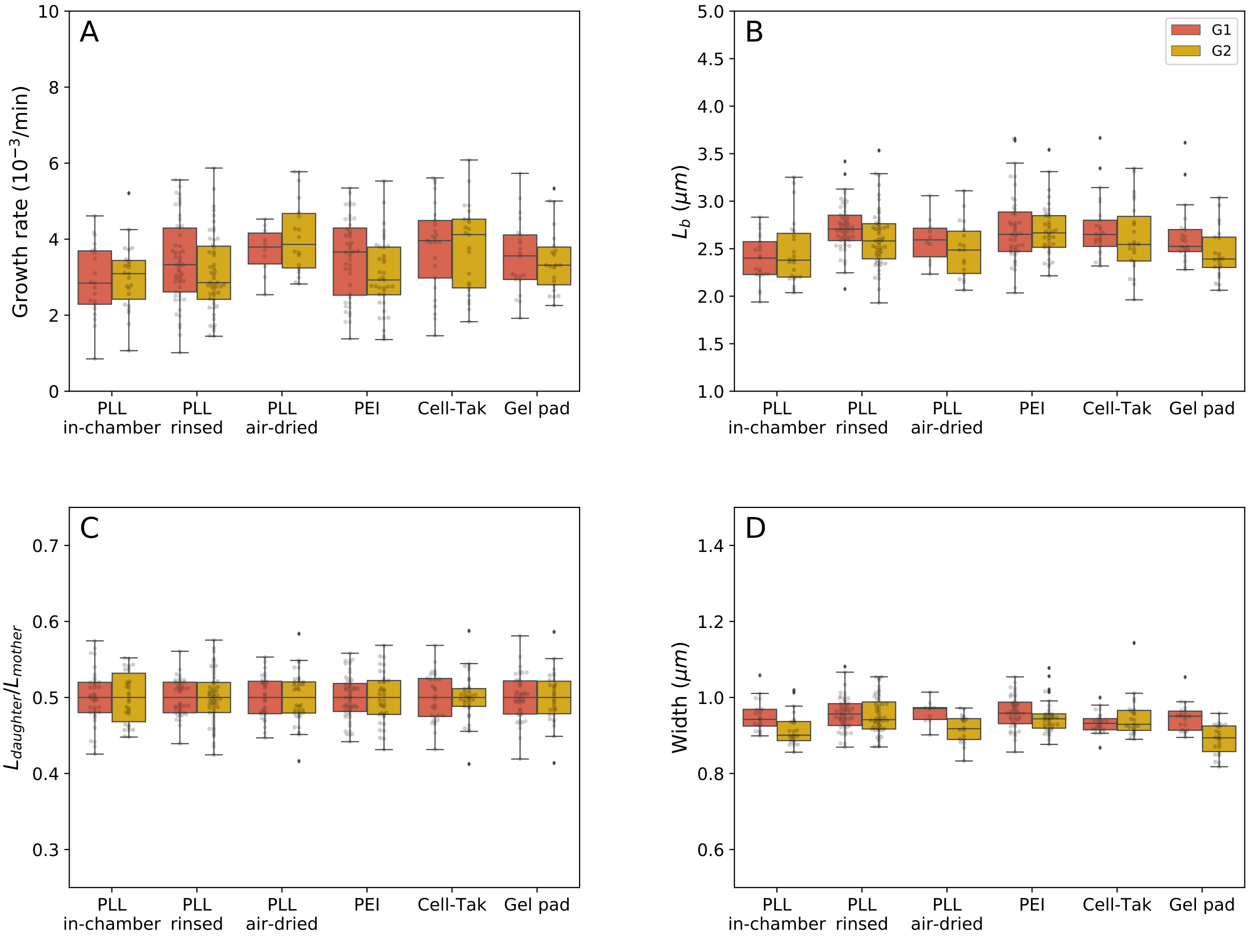

SI Figure 2. Comparison of (**A**) the growth rate, (**B**) initial cell length L_b, (**C**) length ratio $L_{daughter}/L_{mother}$ and (**D**) cell width for G1 and G2 (see Figure 1, top).

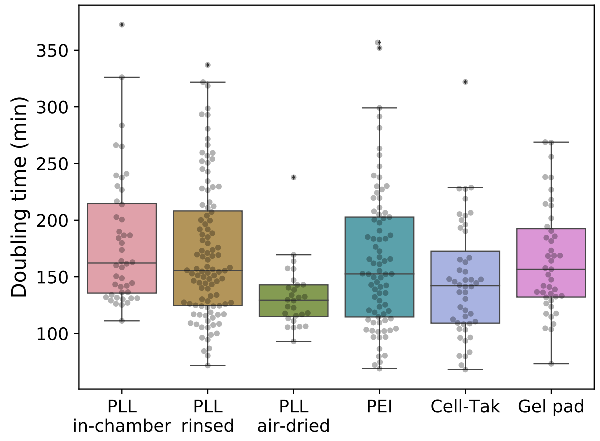

SI Figure 3. Doubling time $t_d = \ln(L_d/L_b)/b$, where b is the individual cell's growth rate.

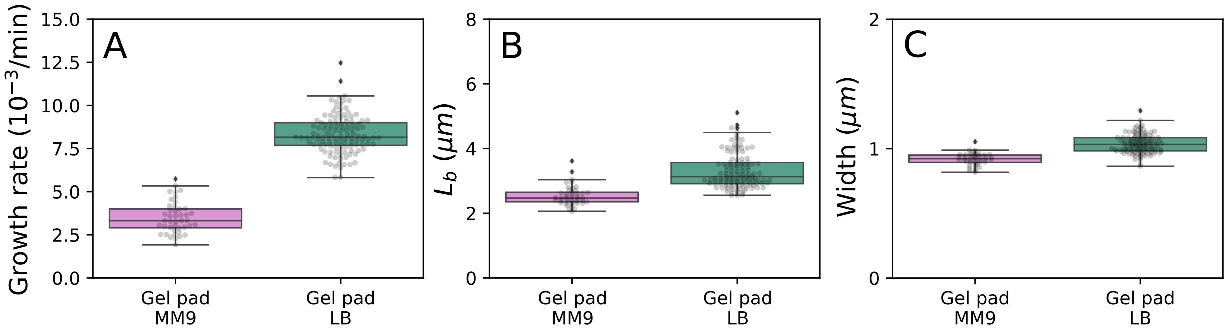

SI Figure 4. Comparison of (**A**) the growth rate (b), (**B**) initial cell length L_b and **(C)** cell width for cells growing on the gel pad in MM9 and LB media.

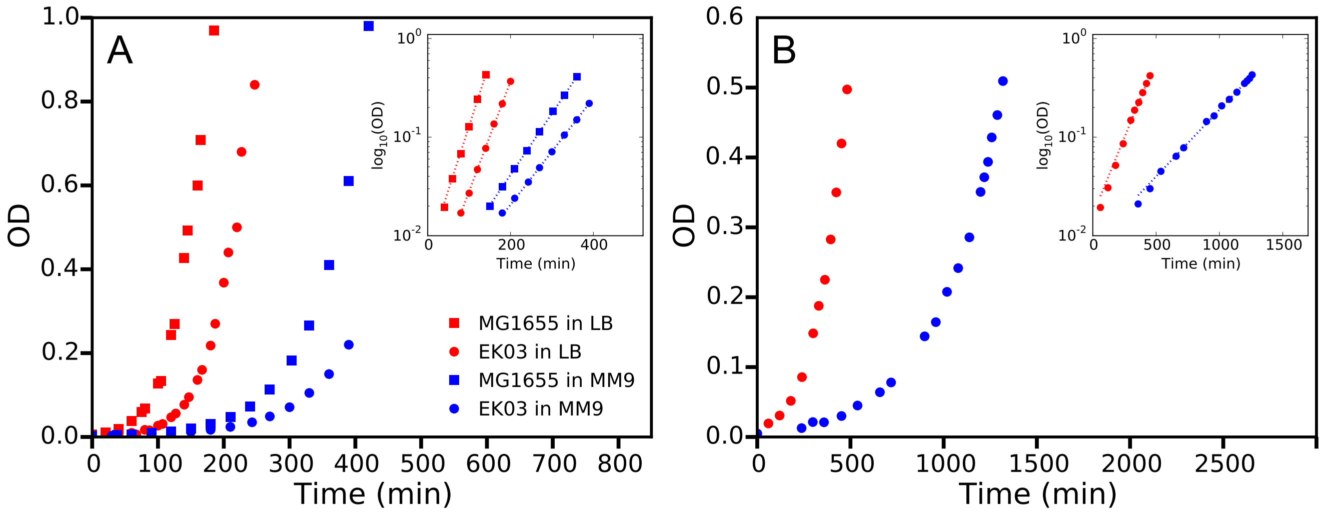

SI Figure 5. Growth curves of MG1655 (squares) and EK03 (circles) strains grown in LB (red) or MM9 (blue) media in the flask at 37 °C (**A**) and 23 °C (**B**) from 10^{-5} starting dilution of the overnight culture. Time scale is renormalised to exclude lag times from the graph, i.e. renormalised $t = 0$ is the time of lowest measurable OD value. Inset: $\log_{10}$(OD) is plotted against time to calculate the growth rates (λ). Dotted lines show exponential fits for OD values between 0.02 and 0.4. See calculated growth rates in SI Table 1.

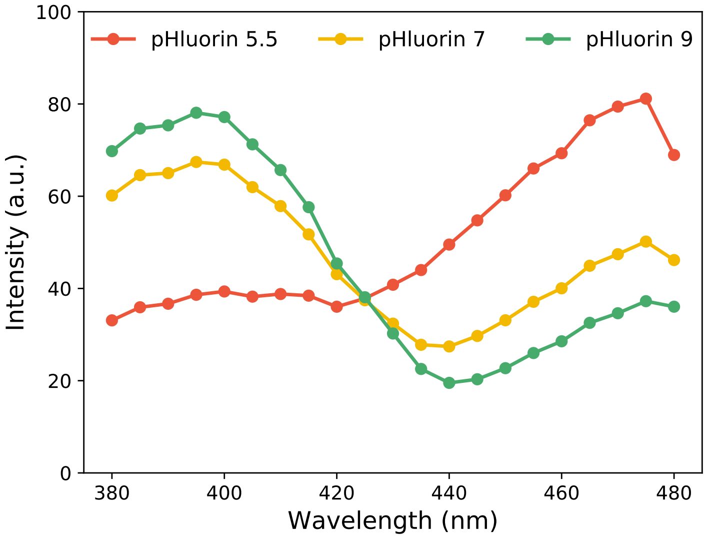

SI Figure 6. Excitation spectra of purified pHluorin at pH values 5.5 (red), 7.0 (yellow) and 9.0 (green). Emission is collected at 510 nm.

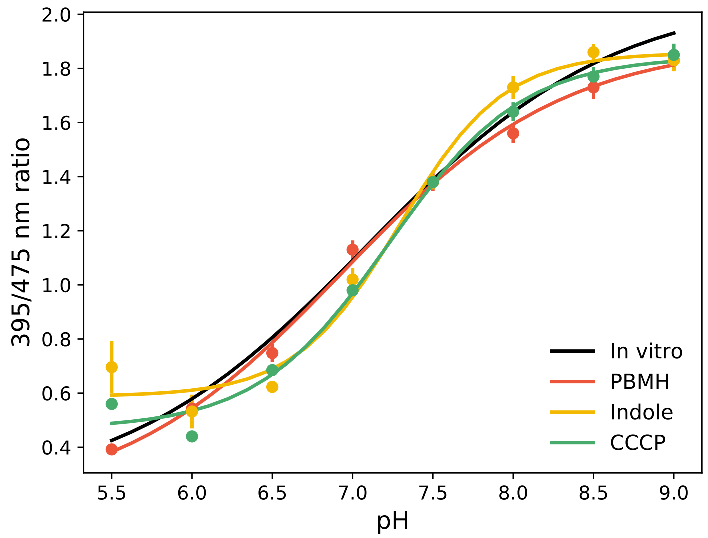

SI Figure 7. Comparison of the *in vivo* and *in vitro* calibration curves. Coloured lines show *in vivo* pHluorin calibration curves with 40 mM PBMH (red), 5 mM indole (yellow) or 25 μM CCCP (green) as ΔpH collapsing agents. Black curve shows *in vitro* calibration curve of purified pHluorin in buffer with no supplements. *In vivo* curve with PMBH aligns best with the *in vitro* curve.

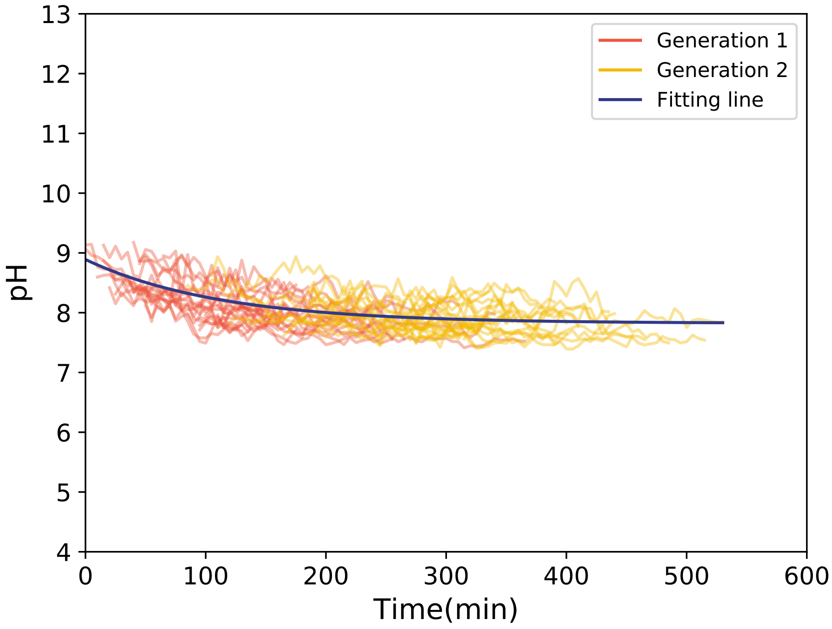

SI Figure 8. Single cell intracellular pH dynamics for two generations of bacteria, first generation is shown in red and the second in yellow. Cells are grown on the PLL ”in-chamber” coated surface. An exponential, shown in blue, was fitted to the whole data set to obtain the final value of pH. The single cell intracellular pH on other coated surfaces shows similar decay.

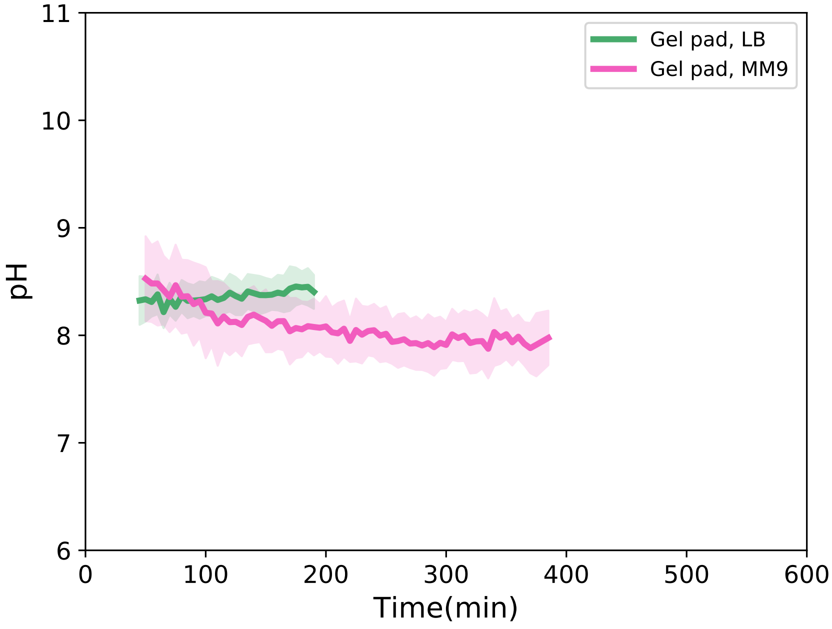

SI Figure 9. Mean and the standard deviation of the intracellular pH of the cells grown in the gel pad in LB (green) or MM9 (red) media.

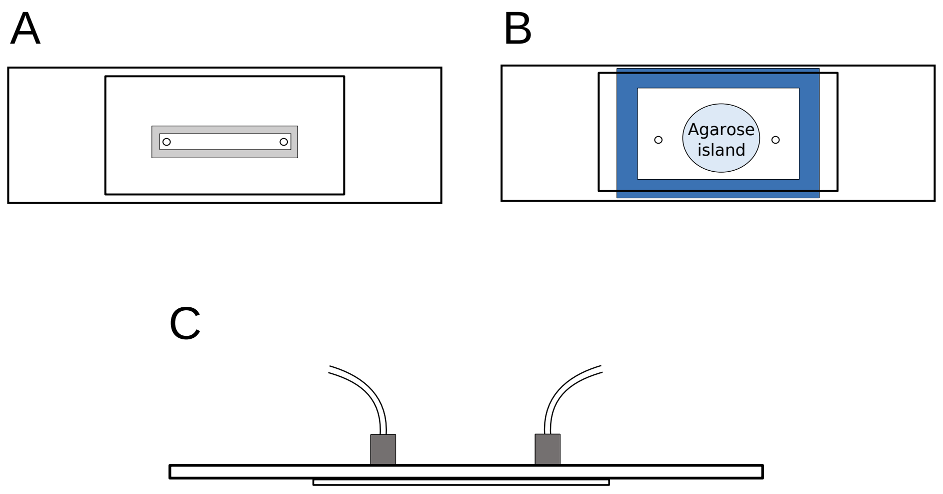

SI Figure 10. Schematic of our flow chambers for bacterial immobilsation experiments. (**A**) top view of the flow chamber used for surface attachments, (**B**) top view of chamber for agarose island experiments, and (**C**) side view of flow chambers.

References

1. Krasnopeeva, E. *Single cell measurements of bacterial physiology traits during exposure to an external stress*. Ph.D. thesis, The University of Edinburgh (2018).

2. Martinez, K. A. *et al.* Cytoplasmic pH response to acid stress in individual cells of Escherichia coli and Bacillus subtilis observed by fluorescence ratio imaging microscopy. *Appl. Environ. Microbiol.* **78**, 3706–3714 (2012). DOI 10.1128/AEM.00354-12.

3. Chimerel, C., Field, C. M., Piñero-Fernandez, S., Keyser, U. F. & Summers, D. K. Indole prevents Escherichia coli cell division by modulating membrane potential. *Biochim. Biophys. Acta - Biomembr.* **1818**, 1590–1594 (2012). DOI 10.1016/j.bbamem.2012.02.022.

4. Krasnopeeva, E., Lo, C.-J. & Pilizota, T. Single-cell bacterial electrophysiology reveals mechanisms of stress-induced damage. *Biophys. J.* **0** (2019). URL https://linkinghub.elsevier.com/retrieve/pii/S0006349519303923http://arxiv.org/abs/1809.05306. DOI 10.1016/j.bpj.2019.04.039. 1809.05306.

5. Schaechter, M., MaalØe, O. & Kjeldgaard, N. O. Dependency on medium and temperature of cell size and chemical composition during balanced growth of salmonella typhimurium. *Microbiol.* **19**, 592–606 (1958). URL https://mic.microbiologyresearch.org/content/journal/micro/10.1099/00221287-19-3-592.

6. Herendeen, S. L., VanBogelen, R. A. & Neidhardt, F. C. Levels of major proteins of escherichia coli during growth at different temperatures. *J. Bacteriol.* **139**, 185–194 (1979). URL https://jb.asm.org/content/139/1/185. https://jb.asm.org/content/139/1/185.full.pdf.

7. Salk, J. E., Youngner, J. S. & Ward, E. N. USE OF COLOR CHANGE OF PHENOL RED AS THE INDICATOR IN TITRATING POLIOMYELITIS VIRUS OR ITS ANTIBODY IN A TISSUE–CULTURE SYSTEM. *Am. J. Epidemiol.* **60**, 214–230 (1954). URL https://doi.org/10.1093/oxfordjournals.aje.a119714. DOI 10.1093/oxfordjournals.aje.a119714. http://oup.prod.sis.lan/aje/article-pdf/60/2/214/622628/60-2-214.pdf.

8. Neidhardt, F., Bloch, P. & Smith, D. F. Culture medium for enterobacteria. *J. bacteriology* **119 3**, 736–47 (1974).
